# Real-Time Communication: Creating a Path to COVID-19 Public Health Activism in Adolescents Using Social Media

**DOI:** 10.2196/21886

**Published:** 2020-12-01

**Authors:** Kunmi Sobowale, Heather Hilliard, Martha J Ignaszewski, Linda Chokroverty

**Affiliations:** 1 Department of Psychiatry and Biobehavioral Sciences University of California Los Angeles Los Angeles, CA United States; 2 Emergency Management Kenner, LA United States; 3 School of Professional Advancement Tulane University New Orleans, LA United States; 4 Department of Psychiatry British Columbia Children’s Hospital Vancouver General Hospital Vancouver, BC Canada; 5 Department of Psychiatry and Behavioral Sciences Albert Einstein College of Medicine Bronx, NY United States

**Keywords:** social media, digital health, COVID-19, adolescent, public health, disaster, communication, affordances

## Abstract

The COVID-19 pandemic and related public health efforts limiting in-person social interactions present unique challenges to adolescents. Social media, which is widely used by adolescents, presents an opportunity to counteract these challenges and promote adolescent health and public health activism. However, public health organizations and officials underuse social media to communicate with adolescents. Using well-established risk communication strategies and insights from adolescent development and human-computer interaction literature, we identify current efforts and gaps, and propose recommendations to advance the use of social media risk communication for adolescents during the COVID-19 pandemic and future disasters.

## Introduction

The COVID-19 pandemic has affected nearly everyone’s way of life, irrespective of their age. The pandemic has particularly challenged adolescents, who at ages 10-19 years are undergoing some of the most dynamic changes of human development [1]. Individuation from family and connection with peers bring fundamental psychological and social benefits to this age group. The loss of in-person social interactions created by stay-at-home mandates, school closures, and social distancing during COVID-19 has strained these developmental processes. Further, because of the protracted nature of the pandemic and its restrictions, pervasive misinformation on the internet, and incorrect perceptions that healthy adolescents are at low risk for infection [2], some young people have dismissed the dangers of infection and their role in transmission in the greater community to spend time with their peers. We are currently witnessing the unsettling crisis of college campuses reopening this fall in the United States, only to face precipitous shutdowns and quarantines in response to outbreaks of COVID-19, brought about by fraternizing young people [3].

Opportunities exist, however, to help counteract some of these challenges during this uncertain time using adolescent-friendly methods. Given the near ubiquitous use of social media (SM) in this age demographic [4], online platforms traditionally used for entertainment purposes can be mobilized to promote health as well. This viewpoint highlights SM’s potential to serve the critical function of protecting and enabling adolescents during this unprecedented time by providing them with accurate and timely health information that will promote individual engagement in healthy behaviors while mitigating risk. Paralleling community adaptation efforts, SM may promote a paradigm shift from passivity and isolationism toward empowerment and activism.

We focus on well-established risk communication strategies and integrate insights from adolescent development and human-computer interaction literatures to identify current efforts, gaps, and recommendations highlighting two main points. First, to adequately support the promotion of healthy behaviors during COVID-19, public health actors should harness the power of SM to address adolescents through their preferred avenues. Second, content must address adolescent-specific needs to activate adolescents as drivers of health protective behaviors and promoters of community adherence.

## Adolescent Use of Social Media

A total of 92% of adolescents use the internet daily and 85% have at least one SM account [4]. Since the start of the COVID-19 pandemic, the use of SM has increased across platforms. For many platforms, particularly those with many adolescent users, the number of new downloads, accounts created, and daily use is at an all-time high [5,6]. In the United States, popular platforms among this age group include YouTube (85%); Instagram (70%); Snapchat (65%) [4]; and, increasingly, TikTok [7]. Because of the ubiquity and stability of adolescent SM use, SM is a promising medium to reach adolescents.

The appeal and frequency of SM use by adolescents can be understood through a developmental perspective. Cognitively, adolescence is marked by a greater ability for self-directed learning and shifts from egocentric thinking to understanding the perspectives of others [8]. In addition to major cognitive changes, adolescence is a time for identity formation and exploration. Socially, adolescents spend more time with peers [9], seeking autonomy from adults. Developmental neuroscience and behavioral science have shown a heightened sensitivity to peer feedback [10,11] and social influences [12] in adolescence. Thus, peer norms and social prestige become more important [1].

The affordances framework provides insight on how SM aligns with these unique aspects of adolescent development [13]. Affordances are key design aspects of SM platforms that underlie how users use them. For adolescents, functional, social, cognitive, emotional, and identity affordances encourage exploration, learning, and practicing of skills that are key for development. Functional affordances refer to features that determine how messages are transmitted and saved for scalability and dissemination, for example, the ability to repost another user’s content. Social affordances are platform features that promote interpersonal interaction. One social affordance is belonging to a group based on a characteristic or shared interest. Identity affordances relate to features allowing users to create and develop their identity and portray it to others. For example, SM profiles can range from platforms that encourage sharing one’s true representation to total anonymity. Cognitive affordances are features that help users learn and share information, for instance, through the use of multimedia such as video as a creative way to promote learning. Emotional affordances are platform features that affect users’ emotional reactions. Examples include the use of personal stories, names, and pictures to create empathy.

Through these affordances, adolescents can explore topics of interest and their identity, connect with others, receive feedback to establish peer norms, and build prestige in a social ecosystem separate from adults. This understanding of adolescent development and the advantages of SM affordance features can inform interventions including risk communication during disasters.

## Risk Communication During Disasters and During COVID-19

Studies have identified risk communication, defined as “the exchange of real time information, advice and opinions between experts and people facing threats to their health, economic or social well-being” [14], as an effective public health intervention to inform the public about resources and promote healthy behaviors during disasters. Increasingly, risk communication occurs through SM platforms due to extensive community reach [15]. SM allows the public, and adolescents in particular, to have timely access to a wide variety of topics with dynamic interaction beyond traditional interface media. At the same time, the inaccuracy of disseminated information may undermine an effective response, which has been unfortunately demonstrated in the context of COVID-19 [16].

Established authorities such as local governments and the Centers for Disease Control and Prevention (CDC) provide messages during public health events by using communication patterns that allow SM users to differentiate true facts from misinformation. The CDC has developed a six-principle framework for the dissemination of information during a crisis called Crisis and Emergency Risk Communications (CERC; Textbox 1) [17]. The CDC developed CERC to enable public health response officials to promptly and effectively communicate with the community in emergency situations. CERC is built around psychological and communication sciences, studies in the field of issues management, and learned experiences [17] from previous emergency responses to allow for communicators to work alongside the media and responders to impact community response and recovery from these potentially devastating emergencies. A full response in a crisis can take time, requiring multiple levels of information to be acquired that is incongruent with the need for the community to respond right away. This delay can impair an adequate community response. This is addressed through the basic principles of CERC, which emphasize the importance of being first, being right, and being credible in disaster response.

**Crisis and Emergency Risk Communications principles [17].**

**Be first**

- Communicating information quickly is crucial. Historically, the first source of information often becomes the preferred source.

**Be right**

- Accuracy establishes credibility. Information can include what is known, what is not known, and actions being implemented to fill in the gaps.

**Be credible**

- Honesty and truthfulness should not be compromised during crises.

**Express empathy**

- Suffering should be acknowledged. Addressing emotional reactions and the challenges faced by individuals leads to trust and rapport.

**Promote action**

- Providing examples of meaningful activities for individuals to engage in may promote calm, reduce anxiety, help restore order, and foster a sense of control [18].

**Show respect**

- Respectful communication promotes cooperation and rapport.

To further promote information sharing, officials from government, health care organizations, and emergency management systems must reach a variety of listeners through their preferred media channels. The implementation of SM for CERC allows for an additional element of interaction to be incorporated into this framework. Whereas traditional news sources release static, precrafted messages in standard formats, such as press releases, the dynamic nature of SM has allowed for shifts in information dissemination. Historically, management agencies or organizers act as senders transmitting information to the receiving community. Through the use of SM, recipients such as adolescents also take on the role of senders by sharing, commenting, or reposting messages to their networks as a functional affordance.

To facilitate the reading and then the sharing of credible health information, content must quickly create resonance with consumers. Given the intentional brevity of SM posts, resonance must be successfully created with concise messages using few characters and through the provision of meaningful messages containing actionable guidance. Because of the rapid turnover of content, online resonance must create “stickiness,” an aspect of functional affordance, so that individuals access and remain on specific content for a greater duration of time. Stickiness can be extended by further engaging audiences with a familiarity of the rules of SM propagation—for example, the use of hashtags to attract readers. Market research and analytics play crucial roles to ensure that content creation and style meet the expectations of the SM user and lead to selective dispersal of accurate information.

In the CERC framework, directives to the community must evolve through the life cycle of a disaster, defined by four phases: preparedness, response, recovery, and mitigation. During these distinct phases the primary needs are concrete and focused on community safety. Nevertheless, some variables remain consistently vital, such as monitoring for hazards and threats, and flexible communication. Messaging about COVID-19 initially focused on reducing the spread of infection by informing, influencing, and empowering readers to wash their hands, stay at home, social distance, and travel only for essential activities such as picking up groceries or prescriptions. As new information has emerged, the current messaging encourages the use of facial masks in public and ongoing promotion of social distancing as communities begin to reduce restrictions and enable reopening.

## Exploring CERC as Applied to Adolescents

Given the pre-existing widespread use of online media and online news sources by the adolescent demographic, CERC messaging through SM may be a particularly effective technique to reach this age group. To effectively engage with adolescents online, messaging must, first, be accurate to be considered an effective public health response. Second, content must be appropriate and relevant to adolescents to be identified as valuable. Here, consideration of the various affordances of SM is important. For adolescents to receive intended communication, it must be posted on platforms that adolescents are routinely accessing. Although the popularity of individual SM platforms will change over time, these affordances will not change and, therefore, can more consistently inform SM interventions. Subsequently, communication on SM that is inclusive, empowering, and promoting appropriate health behaviors can be leveraged as a mechanism to support action by youth and youth activists.

Although national or international health organizations frequently excel in the second and third CERC principles of providing “right” and “credible” information [19], these are time-consuming steps that may prevent adherence to step one of “being first.” By prioritizing speed of information dissemination on SM platforms, organizations and providers may more effectively help adolescents find accurate and up-to-date information from reliable health organizations. Crisis- and disaster-related content directed toward adolescents needs to reflect distilled knowledge from health care providers or public health organizations generated through a funnel process. More simply, this encompasses gathering data, filtering for reliable and consistent information, and then developing content written specifically to promote a selected action. Public health authorities have done little to tailor content on SM platforms for adolescents in prior infectious disease outbreaks [19]. Creating verbiage for adolescent-targeted messaging on SM platforms from health organizations also represents a solution to countering misinformation that has become especially prevalent in the pandemic. In addition, leveraging the functional affordance through ease of replicability of messages and the identity affordance (how adolescents portray themselves online), public health authorities can create precrafted messages for adolescents to repost as their own. This aligns with adolescents’ desire for independence from adults and for prestige through social approval. Further, considering the functional affordance of scalability and the importance of peer norms, collaboration with influencers, especially those of similar age, to spread messages will likely be beneficial.

Available research shows that online misinformation on SM platforms, including during natural disasters, spreads faster and reaches more people than true information [20]. This is particularly troubling with the potential for disinformation to lead to significant harm when accurate information from leading health professionals is delayed or absent [21]. Adolescents may be particularly vulnerable to misinformation, using SM as a primary news source [22]. A recent study demonstrated that 25% of the most popular YouTube videos on COVID-19 contain misinformation [21], and 40%-60% of Twitter posts about COVID-19 are driven by bots (autonomous or semiautonomous software applications that mimic humans) [23]. Health care organizations and other public health agencies are tasked with combating this misinformation through CERC principles. In line with the cognitive affordances of knowledge acquisition and self-efficacy, use of interactive and visual media such as videos or quizzes testing knowledge about COVID-19 with corrective information may be more appealing to adolescents than messages with text or static images.

The next three principles of CERC communication (“express empathy,” “promote action,” “show respect”) are equally as important; these require more specific adaptation for the target demographic and directly address the content of messaging. Language must be selected so as not to downplay information or incite anxiety. Word choice must be within the developmental and cognitive ability of the target demographic. Content must reflect the priorities of adolescents, show empathy for their concerns, and be engaging and entertaining [24].

Highlighting the loss of interactions with friends and community, and missing milestone achievements during the pandemic communicates that adolescent voices are heard in the uncertainty of the pandemic. Adolescents should be encouraged to maintain their connectedness with friends and peers by virtual means in absence of in-person contact when possible (encouragement of physical distancing over social distancing). With support of educational agencies, SM platforms have been used creatively toward managing the somber and disappointing cancellations of important rites of passage and celebrations of achievements such as graduations, proms, and summer job opportunities. For many, these letdowns have been softened and brightened through virtual graduations using livestreaming, video, and photo commemorations of the events in festive attire; distribution of digital diplomas; and inspirational speeches by celebrities and dignitaries [25].

These virtual efforts represent attempts to instill normalcy, highlight shared values, and create a sense of security, comfort, and community. In addition, they exemplify the social affordances of belonging and support, and the emotional affordances of using personal stories and ceremonies to generate emotion and garner empathy [13]. Adolescent struggles should not be minimized but, rather, respectfully validated. These efforts promote an alliance between health providers and organizations and adolescents, and make adolescents feel understood. If they feel camaraderie with the organizations and authorities, adolescents will be more likely to identify the importance of the messages and listen to them.

In regard to promoting action, adolescents have traditionally been seen as passive participants during disasters rather than primary agents and collaborators. Adolescents can play an important role in disaster response and preparedness [26]. Peek and colleagues [27] identified many youth contributions in response to Hurricane Katrina. They found that children and adolescents helped raise and donate money, collect material goods and supplies, assist with rebuilding, support behavioral health, develop programs and raise awareness, and found new organizations. Many youth initiatives were embedded within larger organizational responses rather than independently started by children and adolescents, though the authors acknowledge that adolescent-initiated and encouraged activities are important. A few papers highlight youth’s use of SM to organize volunteering efforts in response to disasters [28,29].

To our knowledge, there have not been any other similar narrative, systematic, gray, or nonscientific literature reviews for adolescent use of SM in response to disasters including infectious disease outbreaks such as COVID-19. Though research in this area is still nascent, SM use aligning with the affordances framework has been identified as a promising medium to promote prevention in adolescents [30], including of sexually transmitted infections [31,32]. A recent systematic review on the use of technology to scale up youth-led participatory action research found few, albeit positive, examples of the powerful nature of adolescent SM engagement [33]. This suggests that adolescents may possess expertise in SM that has been underrecognized and underused, and there is limited documentation of adolescent contributions on SM platforms [27].

This highlights the importance for health care providers to invest in credible communication directed to adolescents and to support their development as emerging and powerful advocates for improving public health behaviors around the world—particularly at this crucial time in public health history. In line with the United Nations’ Sendai Framework for Disaster Risk Reduction 2015-2030 [34] call to include adolescents as agents of change, public health directives should be relayed directly to adolescents.

The best messengers for adolescents are often other adolescents. Some organizations already engage adolescents to participate in response, advocacy, and teaching around important topics in public health. Examples include activation of teen community response teams during disasters (Teen Community Emergency Response Teams [35]) and opportunities for teen leaders to pursue projects that help their communities with preparedness and response during emergencies (the Federal Emergency Management Agency Youth Preparedness Council [36]). Other programs focus on education, empowerment, and building awareness of certain issues (health, youth employment, inequities), accomplished through programs where young people serve as youth ambassadors and youth advocates to the community, such as with Teens Take Charge [37] and the Youth Power Coalition [38]. Enlisting young leaders to promote public health interests allows for this demographic to play an active role in shaping their own future.

Young leaders have already demonstrated great motivation and drive to help others. Content that contains a motivational component can help spread adaptive behaviors to support the community, as reflected through SM posts showing adolescents helping vulnerable neighbors [29]. Social distancing directives in the context of COVID-19 may feel isolating; shifting the schema to highlight the altruism of physical distancing may help to influence desired behaviors at both an individual and population level. This aligns with the adolescents’ desire to portray themselves in a positive light. Further, crafting immediate and accurate messages for both youth engagement as well as activation on SM can promote self-discovery, personal development, and local and global connectedness to perpetuate lasting behavior change [39,40].

Using the right communication model can positively impact behavior and change in adolescents during this pandemic. Leveraging SM is an essential part of the disaster communication toolkit to reach adolescents, who are often missed in targeted communications. Enormous potential exists to harness the existing skills of adolescents and the affordances of SM.

## Current Risk Mitigation Efforts on Social Media

Although there is evidence supporting the use of the CERC framework’s principles during prior disease outbreaks [41,42], the adoption has been slower for adolescents. This may be explained by the fact that many public health and medical organizations previously did not disseminate information through the SM platforms most widely used by youth, such as Instagram, TikTok, Snapchat, and YouTube, or consider affordances [13,22,30,43]. The COVID-19 pandemic has spurred some of these authorities to adapt. For example, the World Health Organization (WHO) started a TikTok account in the wake of COVID-19 and, as of October 2020, has over 2.4 million followers. The CDC has used multiple platforms including YouTube and added a dedicated Snapchat account. The United Nations Children’s Fund (UNICEF) has sought to empower adolescents by asking them to share messages and graphics created by UNICEF and the WHO on SM platforms. More organizations and authorities would benefit from following the example of these pioneer agencies to develop SM accounts that reach a more diversely aged target population, especially where adolescents search and receive health information.

The developers of SM platforms should also be commended for identifying creative methods to promote accurate information. Instagram, Twitter, and TikTok verify authoritative accounts, making it easier for users to identify reputable sources to be featured. Similarly, many platforms have dedicated advertisement space to credible organizations. Many platforms including Facebook, Reddit, Twitter, TikTok, and YouTube now recommend or display credible sources of information associated with certain topics or hashtags and may fact-check content [16,44]. In an effort to correct misinformation, some platforms have displayed videos from credible organizations adjacent to COVID-19 videos with misinformed or unsubstantiated claims; however, this juxtaposition may be confusing to young viewers and lead to the conclusion that videos spreading misinformation are legitimate based on their proximity to videos by subject matter authorities. This is especially concerning because SM content recommendation algorithms can lead to a preferential though unintentional selection of misinformation. SM platforms can also demote, suspend, or even remove accounts spreading misinformation; however, this has been a less popular option due to first amendment rights.

Although organizations have developed accounts on SM to directly address youth, content on SM accounts is often geared toward adults and has not been developed specifically for adolescents or fails to acknowledge the unique challenges they face [22]. Few organizations have addressed the understandable reactions to remote schooling and the loss of sports activities and graduation ceremonies. This is a missed opportunity to promote behavior change through emotional and social affordances. Because empathy and respectful communication build trust, this type of messaging is necessary to engage adolescents. Some examples of successful and empathic ways to engage young people have been demonstrated by the United Nations Educational, Scientific and Cultural Organization (UNESCO), UNICEF, and TikTok. UNESCO’s #MyCOVID-19Story [45] and UNICEF’s #VoicesofYouths [46] campaigns ask youth to describe the challenges their communities face and to share creative and humorous content. TikTok’s livestream event for the graduating class of 2020 featured multiple influencers and allowed students to receive digital diplomas. TikTok’s prom week (#TikTokProm) [47] allowed adolescents to showcase their social distancing prom experience, which generated over 2 billion views. Since the reopening of universities in the United States, some colleges have expanded the role of paid student influencers (hired for marketing their schools to prospective students) to instead model safe behaviors on SM and connect students to resources as part of efforts to contain recent outbreaks on campuses [48].

In addition to empathy, showing respect is likely to activate adolescents. In some cases, adolescents have been admonished or demonized for engaging in risky behavior. Rather than shaming or being condescending, health organizations should provide positive and corrective experiences. Through functional affordance, more efforts should be made to partner with influencers, especially those with large audiences in the target demographic [22]. Indeed, a number of celebrities have streamed conversations with health authorities. Some of these conversations allow users to actively engage by asking questions in real time. For example, users could ask questions on Netflix’s weekly livestream on Instagram called “Wanna Talk About It?” [49], which features conversations between Netflix actors and actresses from popular adolescent shows and mental health professionals to address the mental health and wellness of young people during COVID-19. This interactive way to promote learning and skills exemplifies cognitive affordance.

Many challenges or campaigns have been started to promote action. In general, campaigns try to influence people to engage in a discussion or carry out a set of activities reflecting social affordance. The Vietnamese “Jealous Coronavirus” (Ghen Co Vy) song and the related youth-created #GhenCoVyChallenge dance have been used to promote protective behaviors. The challenge video amassed over 31 million views, and UNICEF and the British Red Cross have shared it [50]. A number of handwashing challenges were created organically and in collaboration with the WHO. The South African Ndlovu Youth Choir created a musical rendition of the WHO’s COVID-19 safety advice shared through SM platforms [51]. Proctor and Gamble hired the top influencer on TikTok, an adolescent 15 years of age with nearly 90 million followers, to perform a dance promoting social distancing labeled the #Distancedance [52]. Further, users were encouraged to create and share their own dance. Snapchat has also leveraged its location-sharing app, Zenly [53], to gamify sheltering-in-place. These interactive and visual risk communication strategies are likely more appealing for adolescents, as they leverage cognitive and social affordances and have generated millions of views and shares.

## Conclusion

Adolescents represent a vulnerable group who are frequently overlooked in disaster and emergency communication. Given the ongoing COVID-19 pandemic and related future challenges of subsequent waves of infection and vaccination, it is a crucial time for risk communication. As explored in this paper, adolescents can be effectively reached and provide positive benefit given their high level of engagement and resourcefulness through online SM platforms. Many global agencies such as UNICEF have highlighted the individual and collective power of adolescent voices emerging as powerful advocates for change, emphasizing the difference between working for adolescents and working with adolescents.

SM can and should be leveraged by health care leaders and public health organizations to access tools to facilitate dynamic, accurate, and actionable risk communication to ensure awareness of public health issues and best practice guidance for adolescents. Nevertheless, this requires adaptation by health authorities to consider the developmental affordances of SM to reach adolescents and to tailor content to adolescent-specific concerns. Though emerging data showcases online connectedness of adolescents and health authorities leads to adherence to safe behavior and civil activism and advocacy, more research is needed to determine the most effective and safest practices as well as the outcomes of such endeavors. We believe that meeting adolescents *where they are at* on SM ensures that voices of young people are heard and represents an investment in their health and well-being during the COVID-19 pandemic and in future disasters.

## Abbreviations

CDC: Centers for Disease Control and Prevention
CERC: Crisis and Emergency Risk Communications
SM: social media
UNESCO: United Nations Educational, Scientific and Cultural Organization
UNICEF: United Nations Children’s Fund
WHO: World Health Organization
